# Objectively Measured Daytime Napping Patterns and All-Cause Mortality in Older Adults

**DOI:** 10.1001/jamanetworkopen.2026.7938

**Published:** 2026-04-20

**Authors:** Chenlu Gao, Ruixue Cai, Xi Zheng, Arlen Gaba, Lei Yu, Aron S. Buchman, David A. Bennett, Lei Gao, Kun Hu, Peng Li

**Affiliations:** 1 Department of Anesthesiology, Mass General Brigham, Harvard Medical School, Boston, Massachusetts; 2 Division of Sleep Medicine, Harvard Medical School, Boston, Massachusetts; 3 Division of Sleep and Circadian Disorders, Brigham and Women’s Hospital, Boston, Massachusetts; 4 Tufts Medical Center, Boston, Massachusetts; 5 Rush Alzheimer’s Disease Center, Rush University Medical Center, Chicago, Illinois; 6 Broad Institute of MIT and Harvard, Cambridge, Massachusetts

## Abstract

**Importance:**

Daytime napping is common among older adults, but evidence linking objective daytime nap patterns to mortality is limited, as prior studies relied largely on self-report. Some daytime nap characteristics, such as timing and variability of nap duration across days, are also understudied.

**Objective:**

To test whether actigraphy-measured daytime nap duration, frequency, variability, and timing are associated with all-cause mortality.

**Design, Setting, and Participants:**

This prospective cohort study used data from the population-based Rush Memory and Aging Project, in which the analytic baseline was initiated in August 2005. Participants in this current study were community-dwelling adults aged 56 years or older in northern Illinois, with up to 19 years of follow-up. Data for this study were gathered until April 2025.

**Main Outcome and Measures:**

Daytime napping, defined as sleeping between 9 am and 7 pm and assessed by up to 14 days of actigraphy data. Cox proportional hazards regression models were used to estimate associations between baseline daytime nap characteristics (duration, frequency, variability in duration across days, and timing) and subsequent all-cause mortality.

**Results:**

Among 1338 participants over a mean (SD) follow-up of 8.30 (4.78) years (mean [SD] participant age, 81.40 [7.39] years; 1018 [76.0%] female), 926 (69.2%) died at a mean (SD) of 7.54 (4.52) years (range, 0.08-19.08 years) after analytic baseline. Participants wore the actigraphy device for a mean (SD) of 9.58 (1.22) days. After adjusting for all covariates, longer daytime nap duration (adjusted hazard ratio [AHR] per 1-hour increase, 1.13; 95% CI, 1.04-1.23; *P* = .005) and higher nap frequency (AHR per additional daily nap, 1.07; 95% CI, 1.02-1.13; *P* = .003) at baseline were associated with increased mortality. Morning nappers had higher mortality risk compared with early afternoon nappers (AHR, 1.30; 95% CI, 1.03-1.64; *P* = .03). Variability in daytime nap duration was not associated with mortality after covariate adjustment (AHR per 1-hour increase, 1.01; 95% CI, 0.89-1.14; *P* = .93).

**Conclusions and Relevance:**

In this prospective cohort study of community-dwelling older adults, longer and more frequent daytime napping, especially in the morning, were associated with higher all-cause mortality. The findings suggest incorporating wearable device–based daytime nap assessments into clinical and public health practices may provide novel opportunities for early risk identification of health conditions.

## Key Points

**Question:**

Are objectively measured daytime nap characteristics, including duration, frequency, variability, and timing, associated with all-cause mortality among community-dwelling older adults?

**Findings:**

In this prospective cohort study of 1338 adults aged 56 years or older, longer and more frequent daytime napping, as well as morning napping, were associated with higher all-cause mortality. Variability in nap duration was not associated with mortality.

**Meaning:**

The findings suggest longer and more frequent, particularly morning, napping may be a behavioral marker of increased mortality risk in late life, underscoring the potential clinical value of incorporating wearable device–based nap assessments into routine health monitoring.

## Teaser

This cohort study of community-dwelling older adults examines whether actigraphy-assessed daytime nap characteristics, including duration, frequency, variability, and timing, are associated with risk of all-cause mortality.

## Introduction

Between 20% and 60% of older adults take naps during the day.^1^ While brief naps can immediately alleviate fatigue and improve alertness,^2^ excessive napping in late life has been linked to adverse health outcomes, including neurodegeneration, cardiovascular diseases, and even greater morbidity.^3,4,5^ In a cohort of older women, those who reported daily napping had a 44% increased mortality risk over 7 years compared with nonnappers.^6^ Similarly, in a British cohort of middle-aged and older adults, self-reported napping for 1 or more hours per day was associated with increased mortality risk.^5^

However, these findings largely relied on self-reported napping habits.^7^ Objective measurements of naps may provide more reliable insights into the relationship between napping and mortality. Because previous studies focused primarily on nap duration and frequency, another gap is the limited consideration of when and how regularly older adults nap.^7^ Morning naps could reflect pronounced sleepiness or circadian rhythm disruption,^8^ whereas naps after lunch may reflect cultural norms (eg, siesta) and align with a natural dip in alertness.^1,9^ Variability in naps across days may indicate fluctuations in underlying health status or contribute to circadian dysregulation, especially in older adults with chronic conditions.^10^ Our group recently showed that variability in nap duration was associated with increased Alzheimer disease pathology at death.^11^ Thus, timing and variability in relation to mortality are crucial aspects of nap behaviors for investigation.

Using data from community-dwelling older adults, we investigated whether objectively measured nap patterns were associated with all-cause mortality. We hypothesized that greater duration, frequency, and variability of naps would be associated with higher mortality risk. We further hypothesized that morning napping would be associated with increased mortality risks compared with afternoon napping.

## Methods

### Participants

In this cohort study, we analyzed data from the Rush Memory and Aging Project (MAP), a clinical-pathologic cohort study initiated in 1997.^12^ Participants were adults recruited from retirement communities, senior and subsidized housing, and church groups in northern Illinois. Race, self-reported using fixed options, were included in the analysis to characterize the cohort and assess the generalizability of findings; categories were American Indian or Alaska Native, Asian, Black or African American, Native Hawaiian or Other Pacific Islander, White, and other (not further specified on the questionnaire) or unknown. Beginning August 2005, wrist actigraphy (Actical [Philips Respironics]) was incorporated as an annual assessment. The analytic baseline was each participant’s first actigraphy assessment. Mortality outcomes were tracked through April 2025. The MAP was approved by an institutional review board of the Rush University Medical Center. All participants signed informed and repository consents as well as the Anatomical Gift Act for organ donation. The current cohort study analyzing MAP data was deemed not human participants research by the institutional review board of Mass General Brigham; thus, informed consent was not required. We followed the Strengthening the Reporting of Observational Studies in Epidemiology (STROBE) reporting guideline for cohort studies.

### Daytime Napping

Participants wore the actigraphy device on their nondominant wrist continuously for up to 14 days. Studies suggest that more than 7 days of actigraphy monitoring yields high reliability in capturing habitual sleep patterns.^13^ The device recorded 3-dimensional acceleration at 32 Hz, which was integrated into 1-dimensional activity counts in 15-second epochs. We resampled the activity count signal into 1-minute epochs and implemented the Cole-Kripke algorithm for sleep detection.^14,15^ To exclude potential off-wrist periods, we removed data segments with 0 activity counts consecutively for 2 or more hours.^4^

Daytime napping was defined as sleep episodes between 9 am and 7 pm.^4^ If 2 sleep episodes were separated by 3 minutes or less, they were merged into 1 episode. We calculated the following variables: nap duration (the mean number of minutes per day spent napping), nap frequency (the mean number of nap episodes per day), and variability in nap duration (the SD of mean nap durations across days for each participant). To characterize nap timing, we identified the 3-hour window with the most naps for each participant (eg, 9 am to 12 pm, 10 am to 1 pm, or 4-7 pm). Participants were categorized as morning nappers (peak window: 9 am to 12 pm or 10 am to 1 pm), early afternoon nappers (peak window: 11 am to 2 pm, 12-3 pm, 1-4 pm, or 2-5 pm), or late afternoon nappers (peak window: 3-6 pm or 4-7 pm).

### All-Cause Mortality and Covariates

Deaths occurring after baseline were ascertained via autopsy records and quarterly contacts. We adjusted for different covariates in 3 sets of models. The basic models adjusted for age, sex, race, and years of education. The intermediate models additionally adjusted for nighttime sleep and circadian rest-activity rhythm at the analytic baseline, including actigraphy-derived total nighttime sleep duration between 9 pm and 7 am, wake after sleep onset, sleep fragmentation, and 2 nonparametric rest-activity rhythm metrics (interdaily stability and intradaily variability). Wake after sleep onset was assessed as the total minutes spent awake between the first and last nighttime sleep epochs. Sleep fragmentation was the likelihood of experiencing an arousal (eg, a nonzero activity count) following an extended period of sleep (approximately 5 minutes).^16^ Interdaily stability reflects the consistency of daily activity rhythm across days. Intradaily variability reflects the fragmentation of daily activity rhythms within a single day.^17^

The full models further adjusted for body mass index, depressive symptoms (Center for Epidemiologic Studies Depression scale),^18^ number of chronic conditions (0-7; included diabetes, heart disease, hypertension, thyroid disease, cancer, head injury, and stroke), medication use (included analgesics, anxiety medications, insomnia medications, anticonvulsants, and β-blockers), physical activity, and disability. Physical activity was the self-reported sum of hours per week spent walking for exercise, gardening or doing yard work, engaging in calisthenics or general exercise, bicycle riding, and swimming or water exercise. Disability was dichotomized by whether participants needed help in any basic activities: walking across a small room, bathing, dressing, eating, getting from bed to a chair, and toileting.

### Statistical Analysis

We used *t* tests and χ^2^ tests to compare characteristics and mortality rates between MAP participants who completed any actigraphy assessment and those who did not. We used Pearson correlations and 1-way analysis of variance (with a Tukey honestly significant difference post hoc test) to test the intercorrelations among baseline nap characteristics and their associations with age.

We used Cox proportional hazards regression models to test associations between each baseline nap variable and mortality. Each nap variable (ie, duration, frequency, variability, and timing) was tested in a separate model. We conducted 3 sets of models, as previously defined in the covariates section. Time to death or the last follow-up assessment in years was included as a continuous variable. To contextualize the magnitude of the effect size, we compared the mortality risk (hazard ratio [HR]) associated with daytime napping with the risk associated with increases in age. For the analyses on nap timing, we excluded participants who napped infrequently (<2 days with naps), napped less than 15 minutes per day, or had multiple nap peak windows to ensure that nap timing classifications reflected habitual rather than incidental or minimal nap patterns.

We conducted 4 sensitivity analyses to ensure the results were not driven by (1) reverse causation (by excluding participants who died within 2 years after baseline); (2) nap outliers (by excluding participants napping >2 hours per day); (3) comorbidity burden (by excluding participants with >2 of the 7 chronic conditions or taking >2 of the 5 medication categories at baseline); or (4) baseline cognitive impairment (by excluding participants with mild cognitive impairment or dementia at baseline), because cognitive impairment was associated with napping in our group’s prior work.^4,11^ Cognitive status was determined by 19 cognitive tests, neuropsychological review, and clinician diagnosis based on National Institute of Neurological and Communicative Disorders and Stroke and Alzheimer’s Disease and Related Disorders Association criteria.

Analyses were conducted in JMP Pro 18 (JMP Statistical Discovery LLC). All tests were 2-tailed, with significance set at *P* < .05. Listwise deletion was used for missing data.

## Results

### Participant Characteristics

Among 1401 participants who completed actigraphy monitoring, we excluded 56 without follow-up or mortality data and 7 with less than 3 days of valid actigraphy. Thus, our analyses included 1338 participants aged 56 years or older, with mean (SD) age of 81.40 (7.39) years; 1018 (76.0%) were female and 320 (24.0%) were male. Three participants (0.2%) were American Indian or Alaska Native; 7 (0.5%), Asian; 76 (5.7%), Black or African American; 1 (0.1%), Native Hawaiian or Other Pacific Islander; 1249 (93.3%), White; and 2 (0.1%), other race. Participants wore the actigraphy device for a mean (SD) of 9.58 (1.22) days.

At baseline, MAP participants who completed actigraphy were younger (*t* = 2.64; *P* = .008), were more likely to be male (χ^2^ = 18.00; *P* < .001), had fewer depressive symptoms (*t* = 4.96; *P* < .001), and reported less disability (χ^2^ = 15.30; *P* < .001) compared with those who did not complete actigraphy, but no differences were found in educational level, race, or the number of medical conditions. The mortality rate was higher among those who completed the actigraphy assessment (χ^2^ = 126.94; *P* < .001).

Most participants (1324 [99.0%]) took naps during the actigraphy period. Distribution of nap variables and analytic baseline characteristics of participants are shown in Table 1. Older participants (with age measured as a continuous variable) had longer nap duration (*r* = 0.25; *P* < .001), higher frequency (*r* = 0.26; *P* < .001), and greater variability in duration (*r* = 0.07; *P* = .01). Early-afternoon nappers were older than morning and late afternoon nappers (*F*_2,1086_ = 11.23; *P* < .001). Nap variables were intercorrelated. Duration was positively correlated with frequency (*r* = 0.93; *P* < .001) and variability (*r* = 0.65; *P* < .001). Frequency was also correlated modestly with variability (*r* = 0.44; *P* < .001). Nap timing was not associated with duration (*F*_2,1086_ = 1.62; *P* = .20) or frequency (*F*_2,1086_ = 1.73; *P* = .18). However, early afternoon nappers had lower variability in nap duration than late afternoon nappers (*F*_2,1086_ = 5.75; *P* = .003).

**Table 1.** Participants’ Characteristics at Analytic Baseline

Characteristic	Participants^a^		
	All (N = 1338)	Survivors (n = 412)	Decedents (n = 926)
Age, mean (SD), y	81.40 (7.39)	76.16 (7.17)	83.73 (6.19)
Sex			
Female	1018 (76.0)	328 (79.6)	690 (74.5)
Male	320 (24.0)	84 (20.4)	236 (25.5)
Race			
American Indian or Alaska Native	3 (0.2)	2 (0.5)	1 (0.1)
Asian	7 (0.5)	3 (0.7)	4 (0.4)
Black or African American	76 (5.7)	42 (10.2)	34 (3.7)
Native Hawaiian or Other Pacific Islander	1 (0.1)	1 (0.2)	0
White	1249 (93.3)	362 (87.9)	887 (95.8)
Other or unknown^b^	2 (0.1)	2 (0.5)	0
Educational level, y	15.06 (3.01)	15.58 (3.15)	14.83 (2.92)
BMI	27.23 (5.37)	28.26 (5.85)	26.75 (5.06)
CESD score for depressive symptoms^c^	0 (0-2)	0 (0-1)	0 (0-2)
Physical activity, h/wk	2.50 (0.75-4.67)	3.00 (1.35-5.50)	2.25 (0.62-4.44)
Chronic conditions, No.^d^	1.61 (1.12)	1.35 (0.98)	1.73 (1.15)
Needs help with basic activities	211 (15.8)	27 (6.6)	184 (19.9)
Medications used			
Analgesic	990 (74.0)	296 (71.8)	694 (74.9)
Antianxiety	90 (6.7)	24 (5.8)	66 (7.1)
Insomnia	110 (8.2)	29 (7.0)	81 (8.7)
Anticonvulsant	144 (10.8)	49 (11.9)	95 (10.3)
β-Blockers	437 (32.7)	103 (25.0)	334 (36.1)
Sleep and circadian rest-activity rhythm			
Nighttime sleep duration, h	4.93 (1.50)	5.14 (1.44)	4.83 (1.52)
Wake after sleep onset, min	129.43 (38.55)	129.35 (37.71)	129.47 (38.94)
Sleep fragmentation index^e^	0.03 (0.02-0.03)	0.03 (0.02-0.03)	0.03 (0.02-0.03)
Interdaily stability	0.52 (0.13)	0.51 (0.12)	0.52 (0.13)
Intradaily variability	1.19 (0.28)	1.12 (0.25)	1.22 (0.29)
Nap behaviors			
Duration, h/d	0.78 (0.36-1.55)	0.72 (0.34-1.48)	0.79 (0.37-1.57)
Frequency, naps/d	1.80 (0.90-3.30)	1.60 (0.83-3.18)	1.90 (0.90-3.40)
Variability in duration across days, h	0.65 (0.37-1.12)	0.67 (0.37-1.22)	0.65 (0.37-1.08)
Timing, No./total No. (%)			
Morning	169/1089 (15.5)	42/330 (12.7)	127/759 (16.7)
Early afternoon	624/1089 (57.3)	176/330 (53.3)	448/759 (59.0)
Late afternoon	296/1089 (27.2)	112/330 (33.9)	184/759 (24.2)

Abbreviations: BMI, body mass index (calculated as weight in kilograms divided by height in meters squared); CESD, Center for Epidemiologic Studies Depression Scale.

^a^ Data are presented as mean (SD) for normally distributed variables, median (IQR) for nonnormally distributed variables, and number (percentage) of participants for categorical variables.

^b^ “Other” was an option on the questionnaire and was not further specified.

^c^ CESD score range, 0 to 9, with higher scores indicating more depressive symptoms.

^d^ Out of 7 total conditions: diabetes, heart disease, hypertension, thyroid disease, cancer, head injury, and stroke.

^e^ Sleep fragmentation index range, 0.02 to 0.08, with higher scores indicating more fragmented sleep.

During up to 19 years (mean [SD], 8.30 [4.78] years) of follow-up, 926 participants (69.2%) died. Mean (SD) time of death was 7.54 (4.52) years (range, 0.08-19.08 years) after analytic baseline. Each additional year increase in age at baseline was associated with an increase in mortality risk (adjusted HR [AHR], 1.12; 95% CI, 1.10-1.13; *P* < .001).

### Nap Duration

Longer nap duration at analytic baseline was associated with increased mortality during follow-up (Table 2). Each 1-hour increase in nap duration was associated with higher mortality in the basic (AHR, 1.24; 95% CI, 1.17-1.31; *P* < .001) and intermediate (AHR, 1.22; 95% CI, 1.14-1.32; *P* < .001) models (full results are shown in eTable 1 in Supplement 1). In the full model, each 1-hour increase in nap duration was also associated with higher risk of mortality (AHR, 1.13; 95% CI, 1.04-1.23; *P* = .005) (Figure, A). Put into context, a 1-hour increase in nap duration corresponded to the risk associated with being approximately 1.1 years older at baseline. The findings were generally consistent in sensitivity analyses, though the associations were attenuated among participants without cognitive impairment (Table 3; full results are shown in eTable 2 in Supplement 1).

**Table 2.** Associations Between Nap Behaviors and All-Cause Mortality Among All Participants

Nap behavior	Model					
	Basic^a^		Intermediate^b^		Full^c^	
	AHR (95% CI)	*P* value	AHR (95% CI)	*P* value	AHR (95% CI)	*P* value
Duration, per 1-h increase	1.24 (1.17-1.31)	<.001	1.22 (1.14-1.32)	<.001	1.13 (1.04-1.23)	.005
Frequency, per additional daily nap	1.13 (1.10-1.17)	<.001	1.10 (1.06-1.15)	<.001	1.07 (1.02-1.13)	.003
Variability in duration across days, per 1-h increase	1.10 (1.00-1.21)	.06	1.03 (0.92-1.15)	.63	1.01 (0.89-1.14)	.93
Timing						
Morning vs early afternoon	1.45 (1.19-1.78)	<.001	1.53 (1.24-1.90)	<.001	1.30 (1.03-1.64)	.03
Morning vs late afternoon	1.42 (1.13-1.78)	.003	1.45 (1.14-1.85)	.002	1.20 (0.92-1.55)	.17

Abbreviation: AHR, adjusted hazard ratio.

^a^ Adjusted for age, sex, educational level, and race.

^b^ Adjusted for all covariates in the basic model plus actigraphy-assessed sleep- and circadian-related variables (nighttime sleep duration, wake after sleep onset, sleep fragmentation index, interdaily stability, and intradaily variability).

^c^ Adjusted for all covariates in the intermediate model plus body mass index, depressive symptoms, physical activity, number of chronic conditions, medication use, and disability.

**Figure.**
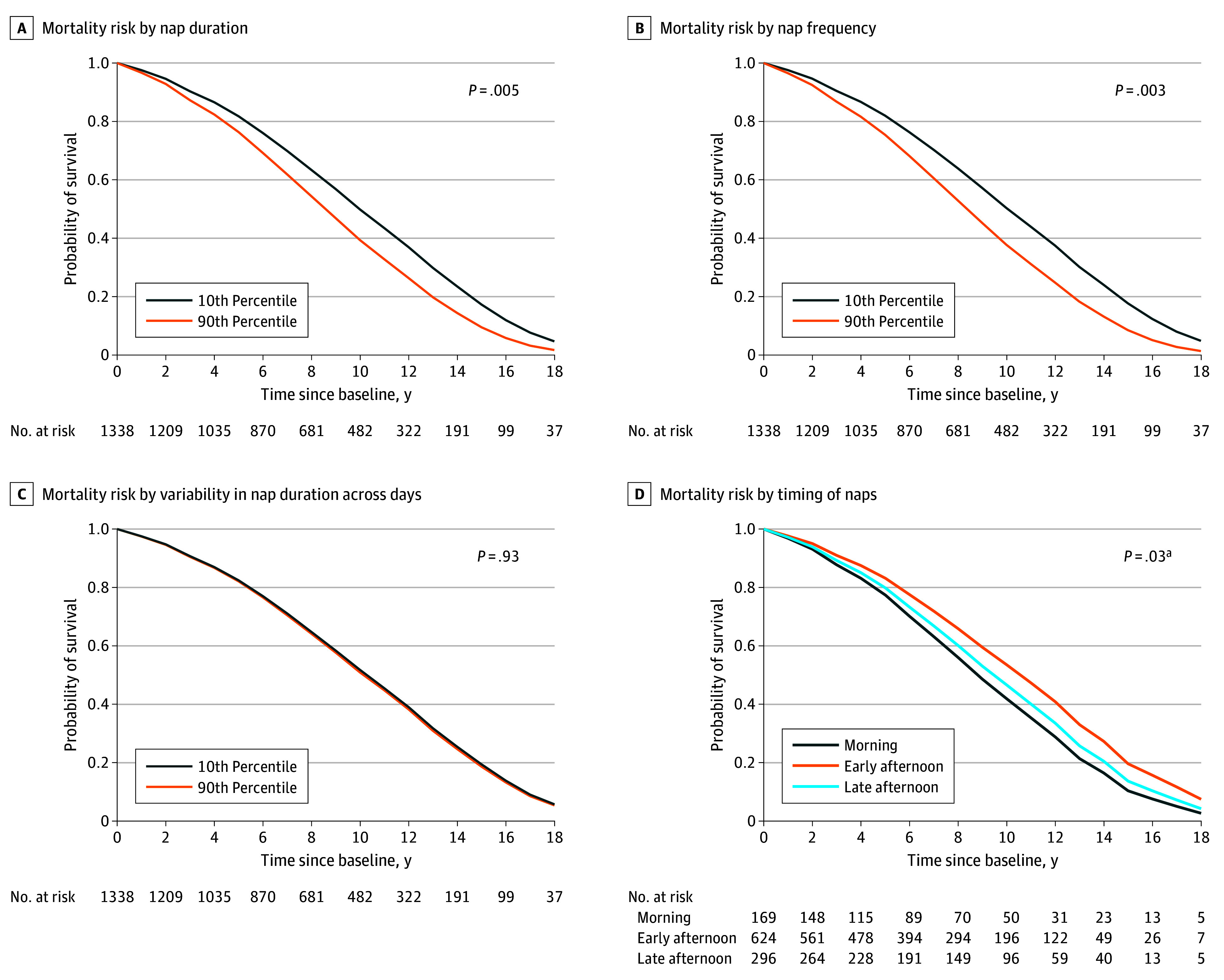
Survival Curves of the Association Between Nap Patterns and Mortality For nap duration, frequency, and variability, lines represent individuals at the 10th and 90th percentile of each measure. Time to death or last follow-up was rounded to the nearest integer (in years). ^a^Comparison for morning and early afternoon groups.

**Table 3.** Associations Between Nap Behaviors and All-Cause Mortality in Sensitivity Analyses

Nap behavior	Sensitivity analysis^a^							
	Excluding participants who died within 2 y (n = 1241)		Excluding participants with daytime sleep duration >2 h/d (n = 1113)		Excluding participants with high comorbidity burden (n = 994)		Excluding participants with cognitive impairment at baseline (n = 943)	
	AHR (95% CI)	*P* value	AHR (95% CI)	*P* value	AHR (95% CI)	*P* value	AHR (95% CI)	*P* value
Duration, per 1-h increase	1.16 (1.05-1.27)	.002	1.24 (1.03-1.50)	.02	1.15 (1.03-1.29)	.01	1.11 (0.99-1.25)	.07
Frequency, per additional daily nap	1.08 (1.03-1.13)	.003	1.11 (1.02-1.21)	.01	1.08 (1.02-1.14)	.004	1.06 (1.00-1.13)	.045
Variability in duration across days, per 1-h increase	1.02 (0.89-1.15)	.81	1.00 (0.87-1.14)	.97	1.02 (0.88-1.18)	.78	0.98 (0.84-1.14)	.78
Timing								
Morning vs early afternoon	1.28 (1.00-1.65)	.049	1.32 (1.02-1.71)	.03	1.38 (1.03-1.84)	.03	1.15 (0.84-1.58)	.38
Morning vs late afternoon	1.22 (0.92-1.61)	.17	1.15 (0.86-1.54)	.34	1.28 (0.93-1.77)	.13	1.07 (0.76-1.50)	.69

Abbreviation: AHR, adjusted hazard ratio.

^a^ Models were adjusted for age, sex, educational level, race, actigraphy-assessed sleep- and circadian-related variables (nighttime sleep duration, wake after sleep onset, sleep fragmentation index, interdaily stability, and intradaily variability), body mass index, depressive symptoms, physical activity, number of chronic conditions, medication use, and disability.

### Nap Frequency

In the basic and intermediate models, each additional daily nap taken at baseline was associated with an increase in mortality risk (basic: AHR, 1.13 [95% CI, 1.10-1.17]; *P* < .001; intermediate: AHR, 1.10 [95% CI, 1.06-1.15]; *P* < .001) (Table 2; full results are shown in eTable 3 in Supplement 1). In the full model, each additional daily nap taken at baseline was also associated with an increase in mortality risk (AHR, 1.07; 95% CI, 1.02-1.13; *P* = .003) (Figure, B), corresponding to the risk associated with being approximately 0.6 years older at analytic baseline. Sensitivity analyses generally yielded consistent results (Table 3; full results are shown in eTable 4 in Supplement 1).

### Nap Variability

Variability in nap duration across days was not significantly associated with mortality risk in any model (AHR per 1-hour increase in the full model, 1.01; 95% CI, 0.89-1.14; *P* = .93) (Figure, C, and Table 2; full results are shown in eTable 5 in Supplement 1). Sensitivity analyses yielded consistent findings (Table 3; full results are shown in eTable 6 in Supplement 1).

### Nap Timing

For analyses of nap timing, we excluded 249 participants (18.6%) who napped infrequently; 37 (14.9%) had less than 2 days with naps, 209 (83.9%) napped less than 15 minutes per day, and 3 (1.2%) had multiple peak nap windows. We examined the association between nap timing and mortality among the remaining 1089 (81.4%) who were habitual nappers. Among them, 759 individuals (69.7%) died, at a mean (SD) of 7.01 (4.28) years (range, 0.08-19.08 years) after analytic baseline. The eFigure in Supplement 1 presents the distribution of daytime nap times, illustrating distinct patterns for morning, early afternoon, and late afternoon nappers and confirming the nap-timing classifications. In the basic model, morning nappers had a higher risk of mortality compared with early afternoon nappers (AHR, 1.45; 95% CI, 1.19-1.78; *P* < .001) and late afternoon nappers (AHR, 1.42; 95% CI, 1.13-1.78; *P* = .003) (Table 2; full results are shown in eTable 7 in Supplement 1). These findings were consistent in the intermediate model (Table 2). In the full model, mortality risk remained elevated only in morning nappers compared with early afternoon nappers (AHR, 1.30; 95% CI, 1.03-1.64; *P* = .03) (Figure, D). This magnitude of risk is equivalent to being approximately 2.5 years older at baseline. In sensitivity analyses, morning nappers continued to exhibit higher mortality risk than early afternoon nappers (Table 3; full results are shown in eTable 8 in Supplement 1). However, when limiting analyses to cognitively intact individuals, there was no significant association between nap timing and mortality.

## Discussion

We found that older individuals with longer nap duration, more frequent naps, and a tendency to nap in the morning were at greater mortality risk over up to 19 years of follow-up. These findings suggest that excessive and morning napping may signal late-life vulnerability.

The high prevalence of napping observed in this cohort is consistent with the established literature indicating that napping is a common behavior among older adults.^1^ Our findings align with a growing body of evidence linking napping with mortality risk.^19,20^ In a dose-response meta-analysis, individuals taking short naps (<1 hour) showed no significant increase in mortality risk, whereas long naps (≥1 hour) were associated with a higher mortality risk.^19,21^ By using multiday actigraphy, our study provided a comprehensive and objective evaluation of multiple nap characteristics and offered novel insights into the napping-mortality association. However, we did not collect concurrent self-reported nap data, and we cannot directly compare the predictive accuracy of actigraphy measures against subjective reports. Compared with duration and frequency, the variability and timing of naps have received less attention. Our study yielded novel insights that early-day naps (when healthy individuals are typically alert) may reflect more underlying health issues.

Cardiovascular pathways are a possible mechanism linking excessive napping to mortality. Sleep disruption and circadian misalignment, which may manifest as excessive napping,^22,23^ can lead to increased blood pressure, attenuated endothelial function, and heightened sympathetic activation.^24^ These changes in autonomic function and vascular system may create a proinflammatory and proatherogenic state that elevates the risk for fatal events. Consistently, past research has associated long daytime naps with cardiovascular risk factors (eg, obesity and high blood pressure) and cardiovascular diseases.^3^ Moreover, excessive daytime sleepiness may be caused by underlying sleep disorders such as obstructive sleep apnea,^25^ which is associated with greater cardiovascular events and mortality.^26^ Notably, our intermediate models demonstrated that the associations between napping and mortality remained significant even after accounting for nighttime sleep duration and quality. These findings suggest that daytime napping is not merely a compensatory response to nocturnal sleep disruption but may serve as an independent marker for mortality risk.

Many chronic health conditions can cause fatigue and excessive sleepiness and prompt napping as a coping mechanism, including chronic lower respiratory diseases, chronic pain, diabetes, cardiovascular conditions,^3^ metabolic syndrome, mood disorders, and neurodegeneration.^4,11^ Although we adjusted for baseline comorbidities, excessive napping may still reflect subclinical or unmeasured conditions that increase both napping and mortality risk. While the underlying mechanisms are complex and vary across conditions, a common feature is that napping is often a compensatory or coping response to disease-related symptoms. Therefore, our findings suggest that excessive napping may be an early marker of underlying health conditions, which ultimately lead to increased mortality if not managed. Contrary to our hypothesis, we did not observe an association between variability in nap duration and mortality. While our group’s previous work showed that nap variability was associated with greater Alzheimer disease pathology, it may be more specific to neurodegeneration than to mortality.^11^ Future research using fractal or nonlinear analysis of rest-activity patterns may capture the complexity of nap irregularity more effectively than SD measurements.

Another possible pathway linking daytime napping to mortality risk is systemic inflammation, which is a well-established risk factor for many chronic diseases and mortality. In observational studies of European middle-aged to older adults, habitual nappers had higher levels of inflammation markers than nonnappers,^27^ suggesting chronic inflammation may induce daytime fatigue and consequently increase napping. Some evidence indicates that the biological underpinnings of inflammation and fatigue can differ by time of day. One study of 543 adult oncology patients showed that morning and evening fatigue were associated with different genetic inflammatory pathways.^28^ Another study of patients with breast cancer reported that morning fatigue was associated with lower body mass index, sleep disturbances, and trait anxiety, whereas evening fatigue was associated with depression.^29^ This evidence supports the idea that morning and afternoon fatigue may arise from distinct underlying conditions, which could explain why the timing of naps has differential health implications.

While we did not examine the specific causes of death, Leng and colleagues^5^ found that excessive nappers had a markedly higher risk of death from respiratory diseases among middle-aged to older British adults. This finding underscores the need for future investigations to assess whether some causes of mortality are more tightly connected to nap patterns, which may help inform the underlying physiologic mechanisms and disease screening. Nevertheless, monitoring changes in a patient’s nap patterns over time may facilitate the identification of individuals who might be at elevated health risk. With the growing use of wearable sleep trackers, there is an opportunity to objectively monitor nap patterns at scale. Integrating wearable device–based measures of naps into routine care or electronic health records could enable clinicians to identify older adults with high daytime sleep burdens and initiate timely evaluations or preventive interventions.

### Limitations

This study has several limitations. First, while actigraphy provides an objective measure of activity and rest, it may not be able to distinguish sleep from quiet wakefulness. We chose the Cole-Kripke algorithm for its relatively high accuracy in detecting daytime sleep.^14,15^ The lack of concurrent self-reported data precludes a direct comparison between subjective and objective nap assessments, limiting our ability to determine if actigraphy offers superior risk prediction compared with self-report. Second, the Rush MAP study sample is predominantly White and precludes the investigation of differences across racial or cultural groups. Napping after lunch (ie, siesta) is a common practice and believed to be prohealth in several cultures, including Hispanic-Latino and Chinese.^1^ Future studies in other racial and ethnic groups are needed to inform the implications of naps in mortality across cultural and racial and ethnic groups. Third, the current observations in older, retired adults may not be generalizable to other age groups or shift workers, as daytime nap patterns could be influenced by age and social or work schedules. Moreover, sleep and napping patterns may vary by season. Larger-scale patterns (eg, by weeks, months, or seasons) in napping patterns and whether they contribute to better identification of individuals with higher mortality risk are yet to be better understood.

## Conclusions

In this prospective cohort study of community-dwelling older adults, we found that actigraphy-assessed longer and more frequent naps, as well as a tendency for morning naps, were associated with greater all-cause mortality. These associations remained robust after adjusting for nocturnal sleep and health factors. Our findings suggest that wearable nap metrics can help identify high-risk individuals and serve as potential targets for rehabilitative or management programs aimed at improving sleep health and longevity in older adults.

## Notes

Supplement 1. **eTable 1.** Association Between Nap Duration and All-Cause Mortality (All Participants) **eTable 2.** Association Between Nap Duration and All-Cause Mortality (Sensitivity Analyses) **eTable 3.** Association Between Nap Frequency and All-Cause Mortality (All Participants) **eTable 4.** Association Between Nap Frequency and All-Cause Mortality (Sensitivity Analyses) **eTable 5.** Association Between Variability in Nap Duration and All-Cause Mortality (All Participants) **eTable 6.** Association Between Variability in Nap Duration and All-Cause Mortality (Sensitivity Analyses) **eTable 7.** Association Between Nap Timing and All-Cause Mortality (All Participants) **eTable 8.** Association Between Nap Timing and All-Cause Mortality (Sensitivity Analyses) **eFigure.** Distribution of Nap Timing

Supplement 2.
Data Sharing Statement
