# Supplementary material for: Objectively Measured Daytime Napping Patterns and All-Cause Mortality in Older Adults
Source: JAMA Netw Open. 2026 Apr 20;9(4):e267938. doi: 10.1001/jamanetworkopen.2026.7938 (PMC13096975; doi:10.1001/jamanetworkopen.2026.7938)
Supplement: Supplement 1. — eTable 1. Association Between Nap Duration and All-Cause Mortality (All Participants) eTable 2. Association Between Nap Duration and All-Cause Mortality (Sensitivity Analyses) eTable 3. Association Between Nap Frequency and All-Cause Mortality (All Participants) eTable 4. Association Between Nap Frequency and All-Cause Mortality (Sensitivity Analyses) eTable 5. Association Between Variability in Nap Duration and All-Cause Mortality (All Participants) eTable 6. Association Between Variability in Nap Duration and All-Cause Mortality (Sensitivity Analyses) eTable 7. Association Between Nap Timing and All-Cause Mortality (All Participants) eTable 8. Association Between Nap Timing and All-Cause Mortality (Sensitivity Analyses) eFigure. Distribution of Nap Timing [file jamanetwopen-e267938-s001.pdf]

Supplementary Online Content

Gao C, Cai R, Zheng X, et al. Objectively measured daytime napping patterns and all-cause mortality in older adults. *JAMA Netw Open*. 2026;9(4):e267938. doi:10.1001/jamanetworkopen.2026.7938

eTable 1. Association Between Nap Duration and All-Cause Mortality (All Participants)

eTable 2. Association Between Nap Duration and All-Cause Mortality (Sensitivity Analyses)

eTable 3. Association Between Nap Frequency and All-Cause Mortality (All Participants)

eTable 4. Association Between Nap Frequency and All-Cause Mortality (Sensitivity Analyses)

eTable 5. Association Between Variability in Nap Duration and All-Cause Mortality (All Participants)

eTable 6. Association Between Variability in Nap Duration and All-Cause Mortality (Sensitivity Analyses)

eTable 7. Association Between Nap Timing and All-Cause Mortality (All Participants)

eTable 8. Association Between Nap Timing and All-Cause Mortality (Sensitivity Analyses)

eFigure. Distribution of Nap Timing

This supplementary material has been provided by the authors to give readers additional information about their work.

© 2026 Gao C et al. *JAMA Network Open*.

eTable 1. Association between nap duration and all-cause mortality (All participants)

	Basic model		Intermediate model		Full model	
	**HR (95% CI)**	**p-value**	**HR (95% CI)**	**p-value**	**HR (95% CI)**	**p-value**
Nap duration (hour)	1.24 (1.17-1.31)	<0.001	1.23 (1.14-1.32)	<0.001	1.13 (1.04-1.23)	0.005
Age	1.12 (1.10-1.13)	<0.001	1.12 (1.10-1.13)	<0.001	1.11 (1.10-1.12)	<0.001
Sex (female)	0.72 (0.62-0.84)	<0.001	0.77 (0.65-0.90)	0.002	0.71 (0.59-0.84)	<0.001
Race (White)	0.98 (0.71-1.36)	0.91	0.97 (0.69-1.36)	0.84	1.04 (0.73-1.48)	0.84
Education (years)	0.96 (0.94-0.98)	<0.001	0.96 (0.94-0.98)	<0.001	0.96 (0.94-0.99)	0.002
Nighttime sleep duration (hours)	--	--	0.98 (0.92-1.04)	0.46	0.97 (0.90-1.04)	0.36
Wake after sleep onset (minutes)	--	--	1.00 (1.00-1.00)	0.01	1.00 (1.00-1.00)	0.11
Sleep fragmentation index[a]	--	--	1.10 (1.02-1.19)	0.01	1.05 (0.96-1.15)	0.26
Interdaily stability	--	--	2.11 (1.10-4.04)	0.02	2.08 (1.04-4.14)	0.04
Intradaily variability	--	--	2.28 (1.66-3.14)	<0.001	2.35 (1.67-3.29)	<0.001
Body mass index (kg/m^2)	--	--	--	--	0.99 (0.97-1.00)	0.06
Depressive symptoms (CESD)	--	--	--	--	1.05 (1.00-1.09)	0.04
Physical activity (hours/week)	--	--	--	--	0.98 (0.95-1.00)	0.04
Number of chronic conditions	--	--	--	--	1.08 (1.01-1.15)	0.03
Analgesic medication (yes)	--	--	--	--	0.83 (0.71-0.98)	0.03
Antianxiety medication (yes)	--	--	--	--	1.06 (0.80-1.40)	0.71
Insomnia medication (yes)	--	--	--	--	1.13 (0.88-1.45)	0.33
Anticonvulsant medication (yes)	--	--	--	--	1.55 (1.21-1.99)	<0.001
Beta blockers (yes)	--	--	--	--	1.13 (0.97-1.32)	0.12
Need help with basic activities (yes)	--	--	--	--	1.76 (1.43-2.15)	<0.001

[a]Variable was standardized. CESD = Center for Epidemiologic Studies Depression Scale.

The Basic model adjusts for age, sex, education, and race/ethnicity.

The Intermediate model adjusts for all covariates in the Basic model, and actigraphy-assessed sleep- and circadian-related variables (nighttime sleep duration, wake after sleep onset, sleep fragmentation index, interdaily stability, intradaily variability).

The Full model adjusts for all covariates in the Intermediate model and body mass index, depressive symptoms, physical activity, number of chronic conditions, medication use, and disability.

© 2026 Gao C et al. *JAMA Network Open*.

eTable 2. Association between nap duration and all-cause mortality (Sensitivity analyses)

	**Sensitivity analysis: Excluding participants died within 2 years**		**Excluding participants with nap > 2 hours/day**		**Excluding participants with high comorbidity burden**		**Excluding participants with cognitive impairment/dementia**	
	**HR (95%CI)**	**p-value**	**HR (95%CI)**	**p-value**	**HR (95%CI)**	**p-value**	**HR (95%CI)**	**p-value**
Nap duration (hour)	1.16 (1.05-1.27)	0.002	1.24 (1.03-1.50)	0.02	1.15 (1.03-1.29)	0.01	1.11 (0.99-1.25)	0.07
Age	1.11 (1.10-1.13)	<0.001	1.11 (1.09-1.12)	<0.001	1.13 (1.11-1.15)	<0.001	1.11 (1.09-1.13)	<0.001
Sex (female)	0.73 (0.60-0.87)	<0.001	0.70 (0.58-0.84)	<0.001	0.72 (0.59-0.89)	0.002	0.69 (0.56-0.86)	<0.001
Race (White)	1.14 (0.77-1.68)	0.51	1.18 (0.79-1.78)	0.42	1.07 (0.68-1.68)	0.77	1.25 (0.75-2.09)	0.40
Education (years)	0.95 (0.93-0.98)	<0.001	0.95 (0.93-0.98)	<0.001	0.98 (0.95-1.01)	0.21	0.96 (0.93-0.99)	0.01
Nighttime sleep duration (hours)	0.96 (0.89-1.03)	0.26	0.99 (0.91-1.08)	0.82	0.95 (0.87-1.04)	0.24	1.00 (0.91-1.09)	0.91
Wake after sleep onset (minutes)	1.00 (1.00-1.00)	0.09	1.00 (1.00-1.00)	0.01	1.00 (1.00-1.00)	0.25	1.00 (0.99-1.00)	0.02
Sleep fragmentation index[a]	1.04 (0.94-1.14)	0.43	1.09 (0.99-1.20)	0.09	1.01 (0.90-1.13)	0.87	1.07 (0.97-1.19)	0.17
Interdaily stability	2.14 (1.03-4.48)	0.04	2.43 (1.11-5.36)	0.03	2.49 (1.08-5.77)	0.03	2.36 (1.01-5.52)	0.05
Intradaily variability	2.19 (1.53-3.13)	<0.001	2.46 (1.68-3.61)	<0.001	1.76 (1.16-2.67)	0.01	2.40 (1.57-3.66)	<0.001
Body mass index (kg/m^2)	0.99 (0.97-1.00)	0.14	0.98 (0.96-1.00)	0.02	0.98 (0.96-1.00)	0.02	0.99 (0.97-1.01)	0.17
Depressive symptoms (CESD)	1.04 (0.99-1.09)	0.11	1.04 (0.99-1.09)	0.15	1.01 (0.96-1.07)	0.62	1.05 (0.99-1.11)	0.13
Physical activity (hours/week)	0.97 (0.95-1.00)	0.04	0.97 (0.95-1.00)	0.03	0.97 (0.95-1.00)	0.05	0.97 (0.94-1.00)	0.04
Number of chronic conditions	1.07 (1.00-1.15)	0.05	1.04 (0.97-1.12)	0.31	1.07 (0.94-1.20)	0.30	1.15 (1.06-1.24)	0.01
Analgesic medication (yes)	0.83 (0.70-0.99)	0.04	0.86 (0.72-1.03)	0.11	0.88 (0.73-1.07)	0.21	0.76 (0.62-0.93)	0.01
Antianxiety medication (yes)	1.02 (0.75-1.38)	0.91	1.14 (0.84-1.54)	0.40	1.20 (0.78-1.84)	0.41	1.00 (0.70-1.44)	0.99
Insomnia medication (yes)	1.15 (0.89-1.48)	0.28	1.23 (0.95-1.60)	0.11	1.19 (0.85-1.67)	0.32	1.08 (0.79-1.46)	0.64
Anticonvulsant medication (yes)	1.57 (1.20-2.05)	0.001	1.62 (1.21-2.17)	0.001	1.95 (1.33-2.86)	<0.001	1.67 (1.22-2.29)	0.002
Beta blockers (yes)	1.09 (0.93-1.29)	0.30	1.20 (1.01-1.42)	0.04	1.16 (0.95-1.41)	0.14	1.13 (0.93-1.37)	0.23
Need help with basic activities (yes)	1.58 (1.26-1.98)	<0.001	1.74 (1.36-2.21)	<0.001	1.94 (1.49-2.53)	<0.001	1.63 (1.25-2.12)	<0.001

[a]Variable was standardized. CESD = Center for Epidemiologic Studies Depression Scale.

The models adjust for age, sex, education, race/ethnicity, actigraphy-assessed sleep- and circadian-related variables (nighttime sleep duration, wake after sleep onset, sleep fragmentation index, interdaily stability, intradaily variability), body mass index, depressive symptoms, physical activity, number of chronic conditions, medication use, and disability.

© 2026 Gao C et al. *JAMA Network Open*.

eTable 3. Association between nap frequency and all-cause mortality (All participants)

	Basic model		Intermediate model		Full model	
	**HR (95% CI)**	**p-value**	**HR (95% CI)**	**p-value**	**HR (95% CI)**	**p-value**
Nap frequency	1.13 (1.10-1.17)	<0.001	1.10 (1.06-1.15)	<0.001	1.07 (1.02-1.13)	0.003
Age	1.12 (1.10-1.13)	<0.001	1.11 (1.10-1.13)	<0.001	1.11 (1.09-1.12)	<0.001
Sex (female)	0.72 (0.62-0.84)	<0.001	0.78 (0.66-0.91)	0.002	0.71 (0.60-0.84)	<0.001
Race (White)	0.98 (0.71-1.37)	0.93	0.98 (0.69-1.37)	0.89	1.06 (0.74-1.51)	0.76
Education (years)	0.96 (0.93-0.98)	<0.001	0.96 (0.94-0.98)	<0.001	0.96 (0.94-0.99)	0.002
Nighttime sleep duration (hours)	--	--	0.98 (0.92-1.05)	0.61	0.96 (0.90-1.04)	0.33
Wake after sleep onset (minutes)	--	--	1.00 (1.00-1.00)	0.01	1.00 (1.00-1.00)	0.11
Sleep fragmentation index[a]	--	--	1.10 (1.01-1.18)	0.02	1.04 (0.95-1.14)	0.37
Interdaily stability	--	--	1.89 (1.00-3.60)	0.05	2.03 (1.03-4.00)	0.04
Intradaily variability	--	--	2.24 (1.63-3.10)	<0.001	2.22 (1.57-3.15)	<0.001
Body mass index (kg/m^2)	--	--	--	--	0.99 (0.97-1.00)	0.06
Depressive symptoms (CESD)	--	--	--	--	1.04 (1.00-1.09)	0.05
Physical activity (hours/week)	--	--	--	--	0.98 (0.95-1.00)	0.04
Number of chronic conditions	--	--	--	--	1.07 (1.00-1.15)	0.04
Analgesic medication (yes)	--	--	--	--	0.83 (0.70-0.98)	0.02
Antianxiety medication (yes)	--	--	--	--	1.06 (0.80-1.40)	0.70
Insomnia medication (yes)	--	--	--	--	1.14 (0.89-1.46)	0.29
Anticonvulsant medication (yes)	--	--	--	--	1.55 (1.21-1.99)	<0.001
Beta blockers (yes)	--	--	--	--	1.14 (0.97-1.32)	0.11
Need help with basic activities (yes)	--	--	--	--	1.79 (1.47-2.19)	<0.001

[a]Variable was standardized. CESD = Center for Epidemiologic Studies Depression Scale.

The Basic model adjusts for age, sex, education, and race/ethnicity.

The Intermediate model adjusts for all covariates in the Basic model, and actigraphy-assessed sleep- and circadian-related variables (nighttime sleep duration, wake after sleep onset, sleep fragmentation index, interdaily stability, intradaily variability).

The Full model adjusts for all covariates in the Intermediate model and body mass index, depressive symptoms, physical activity, number of chronic conditions, medication use, and disability.

© 2026 Gao C et al. *JAMA Network Open*.

eTable 4. Association between nap frequency and all-cause mortality (Sensitivity analyses)

	Sensitivity analysis: Excluding participants died within 2 years		Excluding participants with nap > 2 hours/day		Excluding participants with high comorbidity burden		Excluding participants with cognitive impairment/dementia	
	HR (95% CI)	p-value	HR (95% CI)	p-value	HR (95%CI)	p-value	HR (95%CI)	p-value
Nap frequency	1.08 (1.03-1.13)	0.003	1.11 (1.02-1.21)	0.01	1.08 (1.02-1.14)	0.004	1.06 (1.00-1.13)	0.05
Age	1.11 (1.10-1.13)	<0.001	1.11 (1.09-1.12)	<0.001	1.13 (1.11-1.15)	<0.001	1.11 (1.09-1.13)	<0.001
Sex (female)	0.73 (0.60-0.88)	<0.001	0.70 (0.58-0.84)	<0.001	0.73 (0.59-0.90)	0.003	0.69 (0.55-0.86)	<0.001
Race (White)	1.16 (0.79-1.71)	0.45	1.22 (0.81-1.83)	0.35	1.08 (0.69-1.69)	0.75	1.27 (0.76-2.13)	0.36
Education (years)	0.95 (0.93-0.98)	<0.001	0.95 (0.93-0.98)	<0.001	0.98 (0.95-1.01)	0.21	0.96 (0.93-0.99)	0.01
Nighttime sleep duration (hours)	0.96 (0.89-1.03)	0.25	0.98 (0.89-1.07)	0.63	0.95 (0.87-1.03)	0.22	0.99 (0.91-1.08)	0.87
Wake after sleep onset (minutes)	1.00 (1.00-1.00)	0.10	1.00 (1.00-1.00)	0.01	1.00 (1.00-1.00)	0.28	1.00 (0.99-1.00)	0.02
Sleep fragmentation index[a]	1.03 (0.93-1.13)	0.57	1.07 (0.97-1.18)	0.17	1.00 (0.89-1.11)	0.98	1.06 (0.96-1.18)	0.25
Interdaily stability	2.05 (0.99-4.25)	0.05	2.36 (1.09-5.12)	0.03	2.51 (1.10-5.76)	0.03	2.33 (1.01-5.36)	0.05
Intradaily variability	2.09 (1.45-3.01)	<0.001	2.27 (1.52-3.38)	<0.001	1.71 (1.13-2.59)	0.01	2.30 (1.50-3.53)	<0.001
Body mass index (kg/m^2)	0.99 (0.97-1.00)	0.13	0.98 (0.96-1.00)	0.02	0.98 (0.96-1.00)	0.02	0.99 (0.97-1.01)	0.17
Depressive symptoms (CESD)	1.04 (0.99-1.09)	0.11	1.03 (0.98-1.09)	0.17	1.01 (0.96-1.07)	0.61	1.05 (0.99-1.11)	0.13
Physical activity (hours/week)	0.97 (0.95-1.00)	0.03	0.97 (0.95-1.00)	0.03	0.97 (0.95-1.00)	0.05	0.97 (0.94-1.00)	0.04
Number of chronic conditions	1.07 (1.00-1.15)	0.05	1.04 (0.96-1.11)	0.35	1.06 (0.94-1.20)	0.34	1.15 (1.06-1.24)	<0.001
Analgesic medication (yes)	0.83 (0.70-0.99)	0.04	0.86 (0.72-1.03)	0.10	0.88 (0.73-1.06)	0.18	0.76 (0.62-0.93)	0.01
Antianxiety medication (yes)	1.02 (0.75-1.38)	0.90	1.16 (0.85-1.56)	0.35	1.20 (0.78-1.85)	0.40	1.01 (0.70-1.46)	0.94
Insomnia medication (yes)	1.16 (0.90-1.50)	0.24	1.25 (0.97-1.62)	0.09	1.19 (0.84-1.66)	0.33	1.09 (0.80-1.48)	0.59
Anticonvulsant medication (yes)	1.57 (1.20-2.05)	0.001	1.62 (1.21-2.17)	0.001	1.95 (1.33-2.87)	<0.001	1.65 (1.20-2.26)	0.002
Beta blockers (yes)	1.10 (0.93-1.29)	0.26	1.19 (1.00-1.42)	0.05	1.16 (0.95-1.42)	0.14	1.13 (0.93-1.37)	0.21
Need help with basic activities (yes)	1.62 (1.30-2.03)	<0.001	1.74 (1.37-2.22)	<0.001	1.99 (1.53-2.59)	<0.001	1.65 (1.26-2.15)	<0.001

[a]Variable was standardized. CESD = Center for Epidemiologic Studies Depression Scale.

The models adjust for age, sex, education, race/ethnicity, actigraphy-assessed sleep- and circadian-related variables (nighttime sleep duration, wake after sleep onset, sleep fragmentation index, interdaily stability, intradaily variability), body mass index, depressive symptoms, physical activity, number of chronic conditions, medication use, and disability.

© 2026 Gao C et al. *JAMA Network Open*.

eTable 5. Association between variability in nap duration and all-cause mortality (All participants)

	Basic model		Intermediate model		Full model	
	**HR (95%CI)**	**p-value**	**HR (95%CI)**	**p-value**	**HR (95%CI)**	**p-value**
Variability in nap duration (hour)	1.10 (1.00-1.21)	0.06	1.03 (0.92-1.15)	0.63	1.01 (0.89-1.14)	0.93
Age	1.12 (1.11-1.14)	<0.001	1.12 (1.10-1.13)	<0.001	1.11 (1.10-1.12)	<0.001
Sex (female)	0.72 (0.62-0.84)	<0.001	0.79 (0.67-0.93)	0.01	0.71 (0.60-0.84)	<0.001
Race (White)	0.92 (0.66-1.28)	0.63	0.93 (0.66-1.31)	0.67	1.02 (0.71-1.45)	0.93
Education (years)	0.96 (0.94-0.98)	<0.001	0.96 (0.94-0.98)	<0.001	0.96 (0.94-0.99)	0.004
Nighttime sleep duration (hours)	--	--	1.04 (0.97-1.11)	0.25	1.01 (0.94-1.08)	0.88
Wake after sleep onset (minutes)	--	--	1.00 (1.00-1.00)	0.01	1.00 (1.00-1.00)	0.11
Sleep fragmentation index[a]	--	--	1.15 (1.06-1.24)	<0.001	1.07 (0.98-1.17)	0.15
Interdaily stability	--	--	1.31 (0.69-2.52)	0.41	1.57 (0.79-3.10)	0.20
Intradaily variability	--	--	2.92 (2.14-3.98)	<0.001	2.71 (1.96-3.76)	<0.001
Body mass index (kg/m^2)	--	--	--	--	0.99 (0.97-1.00)	0.07
Depressive symptoms (CESD)	--	--	--	--	1.05 (1.01-1.10)	0.02
Physical activity (hours/week)	--	--	--	--	0.98 (0.95-1.00)	0.05
Number of chronic conditions	--	--	--	--	1.09 (1.02-1.16)	0.01
Analgesic medication (yes)	--	--	--	--	0.84 (0.72-1.00)	0.05
Antianxiety medication (yes)	--	--	--	--	1.04 (0.78-1.38)	0.79
Insomnia medication (yes)	--	--	--	--	1.12 (0.88-1.44)	0.35
Anticonvulsant medication (yes)	--	--	--	--	1.58 (1.24-2.02)	<0.001
Beta blockers (yes)	--	--	--	--	1.17 (1.00-1.36)	0.05
Need help with basic activities (yes)	--	--	--	--	1.80 (1.47-2.20)	<0.001

[a]Variable was standardized. CESD = Center for Epidemiologic Studies Depression Scale.

The Basic model adjusts for age, sex, education, and race/ethnicity.

The Intermediate model adjusts for all covariates in the Basic model, and actigraphy-assessed sleep- and circadian-related variables (nighttime sleep duration, wake after sleep onset, sleep fragmentation index, interdaily stability, intradaily variability).

The Full model adjusts for all covariates in the Intermediate model and body mass index, depressive symptoms, physical activity, number of chronic conditions, medication use, and disability.

© 2026 Gao C et al. *JAMA Network Open*.

eTable 6. Association between variability in nap duration and all-cause mortality (Sensitivity analyses)

	**Sensitivity analysis: Excluding participants died within 2 years**		**Excluding participants with nap > 2 hours/day**		**Excluding participants with high comorbidity burden**		**Excluding participants with cognitive impairment/dementia**	
	**HR (95% CI)**	**p-value**	**HR (95% CI)**	**p-value**	**HR (95%CI)**	**p-value**	**HR (95%CI)**	**p-value**
Variability in nap duration (hour)	1.02 (0.89-1.15)	0.81	1.00 (0.87-1.14)	0.97	1.02 (0.88-1.18)	0.78	0.98 (0.84-1.14)	0.78
Age	1.11 (1.10-1.13)	<0.001	1.11 (1.09-1.12)	<0.001	1.13 (1.11-1.15)	<0.001	1.11 (1.09-1.13)	<0.001
Sex (female)	0.73 (0.60-0.88)	<0.001	0.70 (0.58-0.85)	<0.001	0.74 (0.60-0.91)	0.004	0.69 (0.55-0.85)	<0.001
Race (White)	1.11 (0.76-1.64)	0.59	1.15 (0.76-1.73)	0.51	1.06 (0.67-1.66)	0.81	1.22 (0.73-2.04)	0.45
Education (years)	0.96 (0.93-0.98)	<0.001	0.96 (0.93-0.98)	<0.001	0.98 (0.95-1.01)	0.27	0.96 (0.93-0.99)	0.01
Nighttime sleep duration (hours)	1.00 (0.92-1.08)	0.95	1.02 (0.93-1.11)	0.68	0.99 (0.90-1.08)	0.75	1.03 (0.95-1.12)	0.49
Wake after sleep onset (minutes)	1.00 (1.00-1.00)	0.09	1.00 (1.00-1.00)	0.03	1.00 (1.00-1.00)	0.28	1.00 (0.99-1.00)	0.02
Sleep fragmentation index[a]	1.06 (0.96-1.16)	0.25	1.10 (1.00-1.21)	0.05	1.03 (0.93-1.15)	0.56	1.09 (0.97-1.20)	0.12
Interdaily stability	1.55 (0.75-3.22)	0.24	1.82 (0.84-3.94)	0.13	1.82 (0.79-4.17)	0.16	1.82 (0.78-4.24)	0.16
Intradaily variability	2.56 (1.81-3.63)	<0.001	2.77 (1.90-4.03)	<0.001	2.03 (1.35-3.05)	<0.001	2.69 (1.78-4.05)	<0.001
Body mass index (kg/m^2)	0.99 (0.97-1.00)	0.15	0.98 (0.96-1.00)	0.02	0.98 (0.96-1.00)	0.03	0.99 (0.97-1.01)	0.19
Depressive symptoms (CESD)	1.05 (1.00-1.10)	0.06	1.05 (1.00-1.10)	0.05	1.02 (0.97-1.08)	0.48	1.06 (1.00-1.12)	0.06
Physical activity (hours/week)	0.97 (0.95-1.00)	0.04	0.97 (0.95-1.00)	0.04	0.97 (0.95-1.00)	0.05	0.97 (0.94-1.00)	0.05
Number of chronic conditions	1.09 (1.01-1.16)	0.02	1.06 (0.98-1.14)	0.15	1.07 (0.94-1.20)	0.31	1.17 (1.07-1.26)	<0.001
Analgesic medication (yes)	0.85 (0.71-1.01)	0.07	0.87 (0.73-1.04)	0.14	0.90 (0.74-1.09)	0.28	0.78 (0.63-0.95)	0.01
Antianxiety medication (yes)	1.00 (0.74-1.36)	0.99	1.13 (0.84-1.53)	0.43	1.17 (0.76-1.81)	0.47	0.98 (0.68-1.41)	0.91
Insomnia medication (yes)	1.15 (0.89-1.48)	0.29	1.23 (0.95-1.60)	0.11	1.19 (0.85-1.67)	0.31	1.09 (0.80-1.48)	0.59
Anticonvulsant medication (yes)	1.60 (1.23-2.10)	<0.001	1.64 (1.22-2.19)	0.001	1.96 (1.33-2.87)	<0.001	1.67 (1.22-2.30)	0.001
Beta blockers (yes)	1.13 (0.96-1.33)	0.14	1.23 (1.04-1.46)	0.02	1.18 (0.97-1.44)	0.10	1.16 (0.96-1.41)	0.13
Need help with basic activities (yes)	1.62 (1.30-2.03)	<0.001	1.72 (1.35-2.19)	<0.001	2.02 (1.55-2.63)	<0.001	1.62 (1.24-2.11)	<0.001

[a]Variable was standardized. CESD = Center for Epidemiologic Studies Depression Scale.

The models adjust for age, sex, education, race/ethnicity, actigraphy-assessed sleep- and circadian-related variables (nighttime sleep duration, wake after sleep onset, sleep fragmentation index, interdaily stability, intradaily variability), body mass index, depressive symptoms, physical activity, number of chronic conditions, medication use, and disability.

© 2026 Gao C et al. *JAMA Network Open*.

eTable 7. Association between nap timing and all-cause mortality (All participants)

	Basic model		Intermediate model		Full model	
	HR (95%CI)	p-value	HR (95%CI)	p-value	HR (95%CI)	p-value
Nap timing						
Early afternoon [ref morning]	0.69 (0.56-0.84)	<0.001	0.65 (0.53-0.81)	<0.001	0.77 (0.61-0.97)	0.03
Late afternoon [ref morning]	0.71 (0.56-0.89)	0.003	0.69 (0.54-0.87)	0.002	0.83 (0.64-1.08)	0.17
Morning [ref early afternoon]	1.45 (1.19-1.78)	<0.001	1.53 (1.24-1.90)	<0.001	1.30 (1.03-1.64)	0.03
Late afternoon [ref early afternoon]	1.03 (0.86-1.22)	0.76	1.05 (0.88-1.26)	0.58	1.09 (0.90-1.32)	0.39
Morning [ref late afternoon]	1.42 (1.13-1.78)	0.003	1.45 (1.14-1.85)	0.002	1.20 (0.92-1.55)	0.17
Early afternoon [ref late afternoon]	0.97 (0.82-1.16)	0.76	0.95 (0.79-1.14)	0.58	0.92 (0.76-1.11)	0.39
Age	1.12 (1.11-1.13)	<0.001	1.12 (1.10-1.13)	<0.001	1.11 (1.09-1.12)	<0.001
Sex (female)	0.69 (0.59-0.82)	<0.001	0.77 (0.64-0.92)	0.005	0.68 (0.56-0.82)	<0.001
Race (White)	0.88 (0.62-1.26)	0.49	0.91 (0.63-1.31)	0.60	1.02 (0.70-1.49)	0.93
Education (years)	0.96 (0.94-0.99)	0.003	0.96 (0.94-0.99)	0.004	0.96 (0.94-0.99)	0.01
Nighttime sleep duration (hours)	--	--	1.05 (0.98-1.13)	0.14	1.01 (0.94-1.09)	0.78
Wake after sleep onset (minutes)	--	--	1.00 (1.00-1.00)	0.20	1.00 (1.00-1.00)	0.47
Sleep fragmentation index[a]	--	--	1.14 (1.05-1.24)	0.001	1.07 (0.98-1.18)	0.14
Interdaily stability	--	--	1.23 (0.63-2.41)	0.55	1.57 (0.77-3.18)	0.21
Intradaily variability	--	--	2.81 (2.01-3.92)	<0.001	2.56 (1.79-3.66)	<0.001
Body mass index (kg/m^2)	--	--	--	--	0.98 (0.97-1.00)	0.06
Depressive symptoms (CESD)	--	--	--	--	1.05 (1.00-1.10)	0.05
Physical activity (hours/week)	--	--	--	--	0.97 (0.94-1.00)	0.03
Number of chronic conditions	--	--	--	--	1.07 (0.99-1.15)	008
Analgesic medication (yes)	--	--	--	--	0.81 (0.68-0.97)	0.02
Antianxiety medication (yes)	--	--	--	--	1.04 (0.76-1.42)	0.80
Insomnia medication (yes)	--	--	--	--	1.19 (0.91-1.57)	0.21
Anticonvulsant medication (yes)	--	--	--	--	1.58 (1.22-2.06)	<0.001
Beta blockers (yes)	--	--	--	--	1.15 (0.97-1.36)	0.11
Need help with basic activities (yes)	--	--	--	--	1.96 (1.58-2.44)	<0.001

[a]Variable was standardized. CESD = Center for Epidemiologic Studies Depression Scale.

The Basic model adjusts for age, sex, education, and race/ethnicity.

The Intermediate model adjusts for all covariates in the Basic model, and actigraphy-assessed sleep- and circadian-related variables (nighttime sleep duration, wake after sleep onset, sleep fragmentation index, interdaily stability, intradaily variability).

The Full model adjusts for all covariates in the Intermediate model and body mass index, depressive symptoms, physical activity, number of chronic conditions, medication use, and disability.

© 2026 Gao C et al. *JAMA Network Open*.

eTable 8. Association between nap timing and all-cause mortality (Sensitivity analyses)

	Sensitivity analysis: Excluding participants died within 2 years		Excluding participants with nap > 2 hours/day		Excluding participants with high comorbidity burden		Excluding participants with cognitive impairment/dementia	
	HR (95%CI)	p-value	HR (95%CI)	p-value	HR (95%CI)	p-value	HR (95%CI)	p-value
Nap timing								
Early afternoon [ref morning]	0.78 (0.61-1.00)	0.05	0.76 (0.58-0.98)	0.03	0.73 (0.54-0.97)	0.03	0.87 (0.63-1.19)	0.38
Late afternoon [ref morning]	0.82 (0.62-1.09)	0.17	0.87 (0.65-1.16)	0.34	0.78 (0.56-1.07)	0.13	0.93 (0.67-1.31)	0.69
Morning [ref early afternoon]	1.28 (1.00-1.65)	0.05	1.32 (1.02-1.71)	0.03	1.38 (1.03-1.84)	0.03	1.15 (0.84-1.58)	0.38
Late afternoon [ref early afternoon]	1.06 (0.86-1.29)	0.60	1.15 (0.93-1.42)	0.20	1.07 (0.85-1.34)	0.55	1.08 (0.85-1.36)	0.53
Morning [ref late afternoon]	1.22 (0.92-1.61)	0.17	1.15 (0.86-1.54)	0.34	1.28 (0.93-1.77)	0.13	1.07 (0.76-1.50)	0.69
Early afternoon [ref late afternoon]	0.95 (0.77-1.16)	0.60	0.87 (0.70-1.08)	0.20	0.93 (0.74-1.17)	0.55	0.93 (0.74-1.17)	0.53
Age	1.11 (1.09-1.13)	<0.001	1.10 (1.08-1.12)	<0.001	1.13 (1.11-1.15)	<0.001	1.11 (1.09-1.13)	<0.001
Sex (female)	0.69 (0.56-0.85)	<0.001	0.66 (0.53-0.82)	<0.001	0.70 (0.55-0.88)	0.003	0.64 (0.50-0.82)	<0.001
Race (White)	1.14 (0.74-1.73)	0.56	1.20 (0.77-1.88)	0.43	0.92 (0.57-1.49)	0.74	1.19 (0.68-2.06)	0.54
Education (years)	0.95 (0.93-0.98)	0.002	0.95 (0.93-0.98)	0.002	0.98 (0.95-1.01)	0.21	0.96 (0.92-0.99)	0.01
Nighttime sleep duration (hours)	1.00 (0.92-1.09)	0.97	1.02 (0.93-1.12)	0.61	1.00 (0.90-1.10)	0.96	1.03 (0.94-1.13)	0.52
Wake after sleep onset (min)	1.00 (1.00-1.00)	0.43	1.00 (1.00-1.00)	0.20	1.00 (1.00-1.00)	0.80	1.00 (0.99-1.00)	0.07
Sleep fragmentation index[a]	1.05 (0.95-1.16)	0.33	1.10 (0.99-1.22)	0.06	1.04 (0.91-1.16)	0.56	1.08 (0.96-1.20)	0.18
Interdaily stability	1.47 (0.69-3.14)	0.32	1.77 (0.78-4.00)	0.17	1.71 (0.71-4.13)	0.23	1.92 (0.80-4.61)	0.15
Intradaily variability	2.36 (1.61-3.47)	<0.001	2.78 (1.81-4.25)	<0.001	1.91 (1.22-2.98)	0.005	2.41 (1.51-3.84)	<0.001
Body mass index (kg/m^2)	0.99 (0.97-1.00)	0.12	0.98 (0.96-0.99)	0.01	0.97 (0.95-0.99)	0.01	0.99 (0.97-1.00)	0.14
Depressive symptoms (CESD)	1.05 (0.99-1.10)	0.09	1.04 (0.99-1.10)	0.12	1.02 (0.96-1.08)	0.60	1.06 (0.99-1.12)	0.07
Physical activity (hours/week)	0.97 (0.94-0.99)	0.02	0.96 (0.94-0.99)	0.02	0.97 (0.94-1.00)	0.03	0.97 (0.93-1.00)	0.06
Number of chronic conditions	1.06 (0.98-1.15)	0.12	1.02 (0.94-1.11)	0.59	1.03 (0.90-1.18)	0.69	1.15 (1.05-1.26)	0.003
Analgesic medication (yes)	0.80 (0.66-0.97)	0.03	0.81 (0.66-1.00)	0.05	0.86 (0.70-1.07)	0.17	0.70 (0.56-0.88)	0.003
Antianxiety medication (yes)	1.00 (0.71-1.41)	0.99	1.15 (0.82-1.62)	0.41	1.19 (0.73-1.94)	0.49	0.96 (0.64-1.44)	0.84
Insomnia medication (yes)	1.21 (0.91-1.61)	0.18	1.38 (1.03-1.85)	0.03	1.20 (0.81-1.76)	0.36	1.20 (0.85-1.70)	0.31
Anticonvulsant medication (yes)	1.61 (1.21-2.14)	0.001	1.68 (1.23-2.31)	0.001	1.77 (1.16-2.69)	0.01	1.78 (1.27-2.49)	<0.001
Beta blockers (yes)	1.10 (0.92-1.32)	0.29	1.25 (1.03-1.52)	0.02	1.17 (0.94-1.46)	0.15	1.14 (0.92-1.41)	0.24
Need help with basic activities (yes)	1.80 (1.41-2.29)	<0.001	1.98 (1.52-2.59)	<0.001	2.18 (1.65-2.88)	<0.001	1.88 (1.40-2.52)	<0.001

[a]Variable was standardized. CESD = Center for Epidemiologic Studies Depression Scale.

The models adjust for age, sex, education, race/ethnicity, actigraphy-assessed sleep- and circadian-related variables (nighttime sleep duration, wake after sleep onset, sleep fragmentation index, interdaily stability, intradaily variability), body mass index, depressive symptoms, physical activity, number of chronic conditions, medication use, and disability.

© 2026 Gao C et al. *JAMA Network Open*.

eFigure. Distribution of nap timing

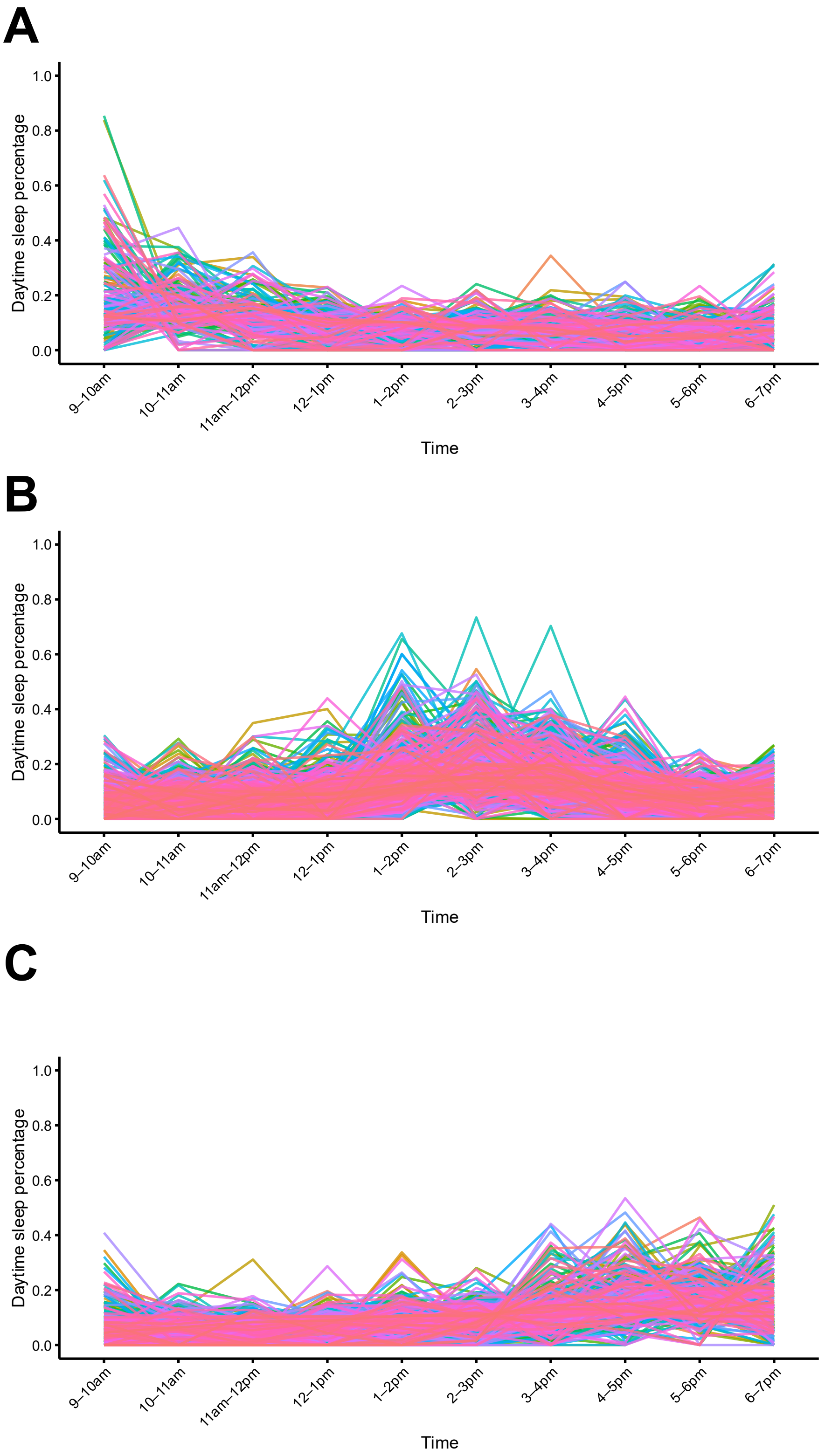

The distribution of naps during each hour for morning nappers (A), early afternoon nappers (B), and late afternoon nappers (C). The percentage of nap for each hour was calculated by dividing the total minutes of naps during that hour by the total minutes of naps between 9 a.m. and 7 p.m. Colors of the lines are used for the visual differentiation of individual participant trajectories and do not represent specific subgroups.

© 2026 Gao C et al. *JAMA Network Open*.
