# Supplementary material for: Objectively Measured Daytime Napping Patterns and All-Cause Mortality in Older Adults
Source: JAMA Netw Open. 2026 Apr 20;9(4):e267938. doi: 10.1001/jamanetworkopen.2026.7938 (PMC13096975; doi:10.1001/jamanetworkopen.2026.7938)
Supplement: Supplement 2. — Data Sharing Statement [file jamanetwopen-e267938-s002.pdf]

Data Sharing Statement

Gao. Objectively Measured Daytime Napping Patterns and All-Cause Mortality in Older Adults. *JAMA Netw Open*. Published April 20, 2026. doi:10.1001/jamanetworkopen.2026.7938

Data
Data available: No

Additional Information
Explanation for why data not available: The data are available under restricted access from the Rush Alzheimer's Disease Center (RADC) following the data and resource sharing policy. Access of data can be obtained by submitting requests through the RADC Resource Hub at www.radc.rush.edu.
